# Supplementary material for: Management of E. coli sister chromatid cohesion in response to genotoxic stress
Source: Nat Commun. 2017 Mar 6;8:14618. doi: 10.1038/ncomms14618 (PMC5343486; doi:10.1038/ncomms14618)
Supplement: Supplementary Information — Supplementary Figures, Supplementary Tables, and Supplementary Methods and Supplementary References [file ncomms14618-s1.pdf]

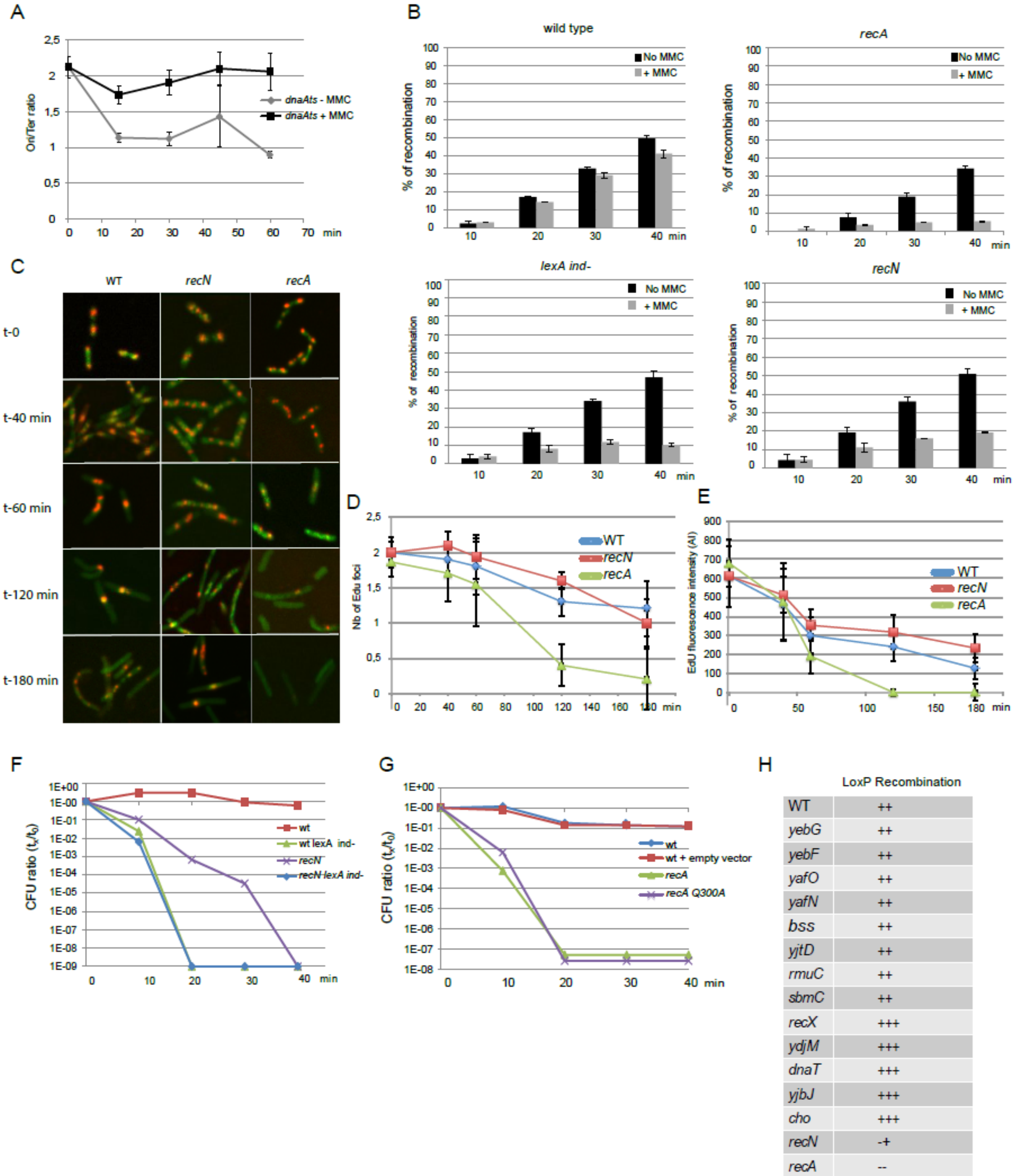


H

LoxP Recombination

| | |
|---|---|
| WT | ++ |
| *yebG* | ++ |
| *yebF* | ++ |
| *yafO* | ++ |
| *yafN* | ++ |
| *bss* | ++ |
| *yjtD* | ++ |
| *rmuC* | ++ |
| *sbmC* | ++ |
| *recX* | +++ |
| *ydjM* | +++ |
| *dnaT* | +++ |
| *yjbJ* | +++ |
| *cho* | +++ |
| *recN* | -+ |
| *recA* | -- |

## Supplementary Figure 1

**A)** MMC provokes a rapid replication arrest. We used a strain with a *dnaA*ts allele to block the initiation of replication when placed at 40°C. The *ori/ter* ratio was monitored by qPCR. In the repressive condition the progression of replication provokes an increase of the amount of terminus region. In the presence of MMC the *ori/ter* ratio is constant, suggesting replication arrest. **B)** Frequency of *loxP*/Cre recombination at the ori-3 locus for various mutants. *LoxP* assays were performed 10, 20, 30 and 40 min after addition of 10µg/ml MMC. *LoxP* recombination frequency was monitored in the WT, *recA*, *lexA ind-*

(SOS down) and *recN* mutants treated with MMC (10µg/ml) or not. The frequency of recombination products (1 +3 *loxP*) compared to non recombined products (2 *loxP*) was measured by PCR and bioanalyzer detection. Results are expressed as the *loxP* recombination frequency at each timepoint (1+3*loxP)*/(1+2+3*loxP*). Error bars are standard deviation of 4 experiments. **C)** Monitoring degradation of newly replicated DNA in the WT, *recN* and *recA* mutants. EdU was incorporated for 10 min before addition of MMC, at the indicated time-point, after addition of MMC, the cells were washed, fixed and immuno-stained (red), DNA was stained with DAPI (green). **D)** Quantification of the average number of EdU foci per nucleoids of the cells presented in panel C. **E)** Quantification of the average intensity of EdU fluorescence in the cells presented in panel C. **F)** Cell viability in response to MMC (10µg/ml) for the indicated mutants. Bacteria were treated for the indicated amount of time with MMC then washed and plated on LB plates. The data are plotted as a ratio between the number of colonies formed in the absence of MMC and the number of colonies formed in the presence of MMC. Error bars are standard deviation of 200 cells. **G)** Cell viability in response to MMC (10µg/ml) for the indicated mutants. Experiments were performed as in Fig S1G. **H)** *loxP* recombination in response to 10µg/ml MMC treatment for various SOS mutants. Mutants were treated for 40 min with 10µg/ml MMC or not. Results are expressed as the mutant recombination frequency with MMC over the wild type recombination frequency with MMC.

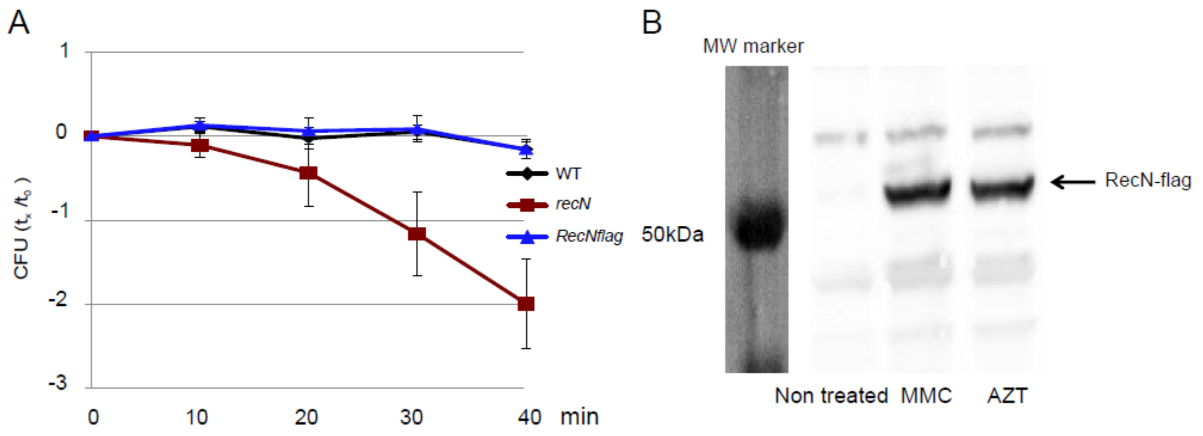


**Supplementary Figure 2**

**A)** Cell viability of the RecN flag-tagged strain compared to the WT and *recN* strains. Cells were treated for 10, 20, 30 or 40 min with 10µg/ml MMC. Cell viability was assessed for each time point. Error bars are standard deviation of 3 experiments. **B)** RecN protein is expressed in cells treated with 10µg/ml MMC or 1µg/ml AZT. Western blot analysis on a RecN-flag protein reveals that RecN protein is induced in response to a 40 min MMC treatment or a 40 min AZT treatment. RecN is strongly repressed in the absence of DNA damage. Secondary antibody coupled with Horse radish peroxidase was used to reveal RecN-flag protein. The MW marker lane was acquired using bright field light.

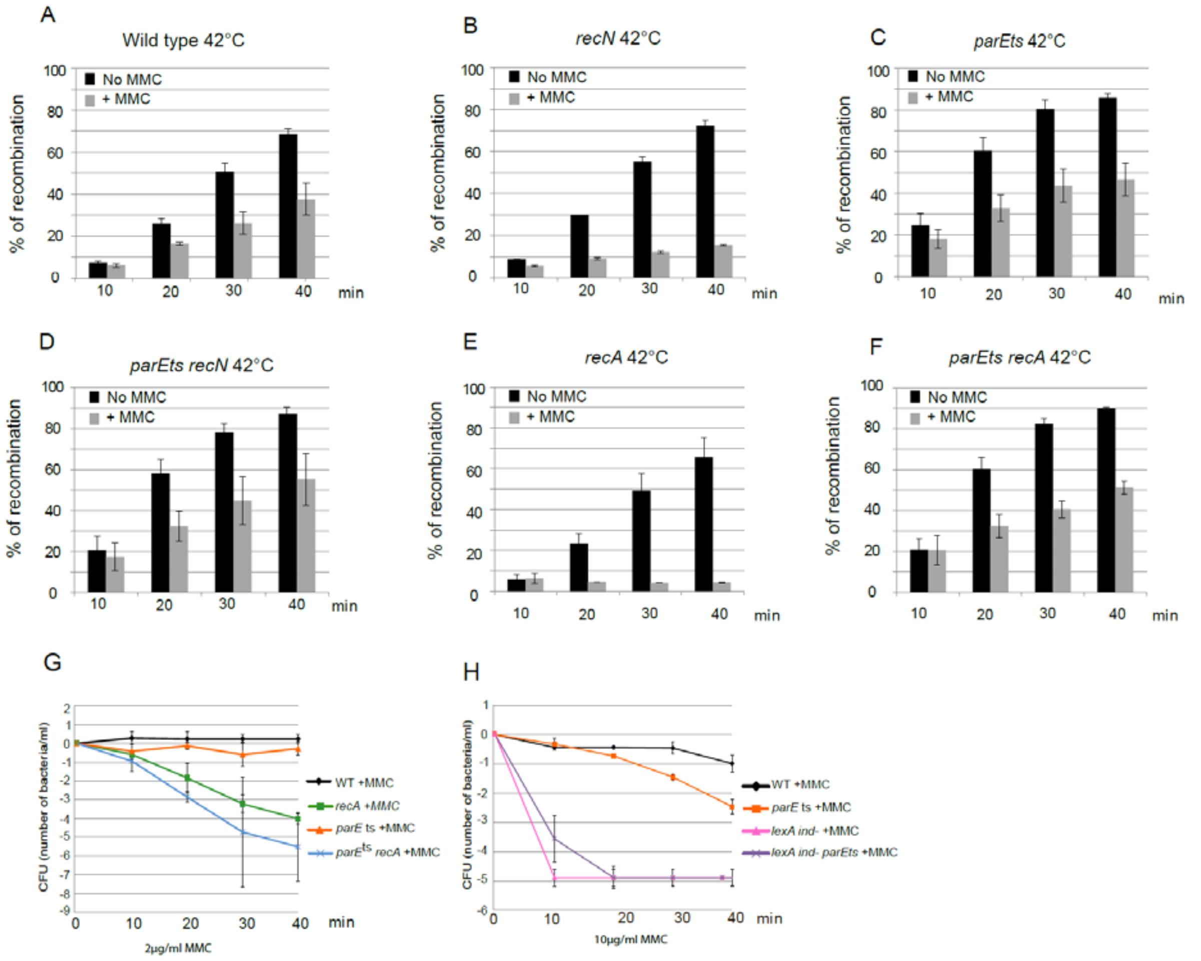


**Supplementary Figure 3**

**A-F)** Measure of SCIs following TopoIV alterations in the presence of MMC. *loxP* assays were performed 10, 20, 30 and 40 min after addition of 10µg/ml MMC. Results are expressed as the *loxP* recombination frequency for each timepoint. The cells were incubated 25min at 42°C prior to addition of arabinose (for Cre induction) and MMC (10µg/ml). Error bars are standard deviation of 3 experiments. **G)** Cell viability in response to MMC (2µg/ml) for the indicated mutants. **H)** Cell viability in response to MMC (10µg/ml) for the indicated mutants. Error bars are standard deviation of 3 experiments.

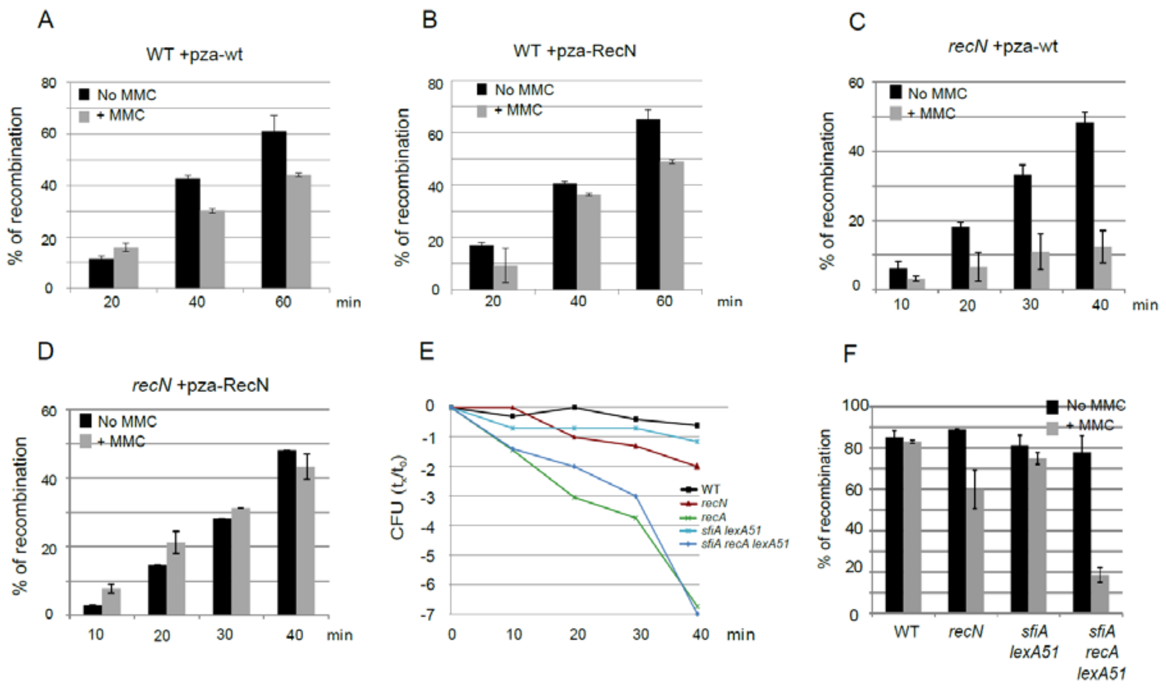


**Supplementary Figure 4**

**A-D)** Influence of *RecN* overexpression on SCIs. *recN* was cloned onto a plasmid with a leaky promoter. LoxP recombination frequency was measured at 10, 20, 30 and 40min. Results are expressed as the percentage of *loxP* recombination at each time point. **E)** Cell viability of constitutively induced SOS strains. Cells were treated for 10, 20, 30 or 40min with 10µg/ml MMC. Cell viability was assessed for each time point. **F)** SCIs of constitutively induced SOS strains. Cells were treated for 40min with 10 µg/ml of MMC. Due to poor growth conditions in minimum medium, the assay was performed in LB. Results are expressed as the percentage of *loxP* recombination at each time point. Error bars are standard deviation of 3 experiments.

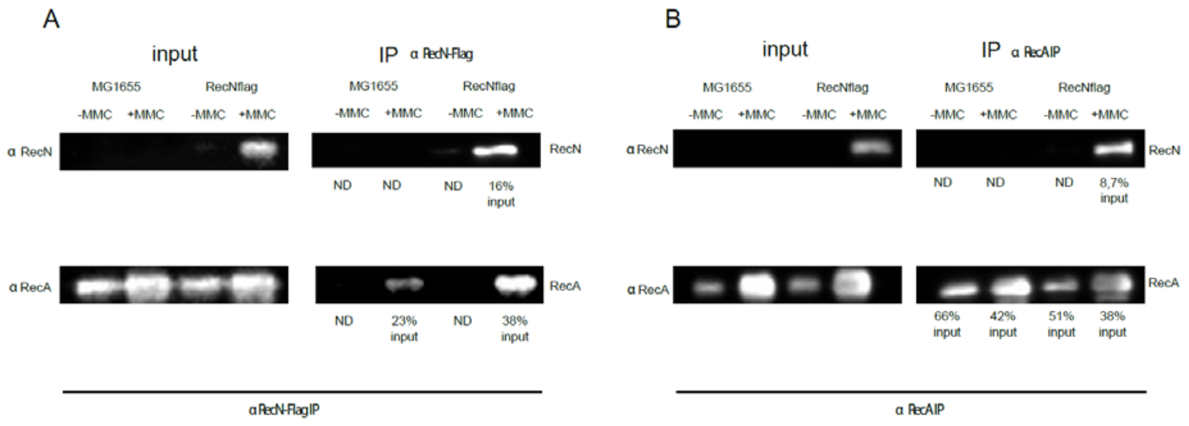


**Supplementary Figure 5**

**A)** RecA co-immunoprecipitates with RecN. Immunoprecipitation with an anti-flag antibody was done on the RecN protein tagged with a flag peptide and a WT MG1655 strain. Samples were blotted with an anti-flag antibody (top panel) or an anti-RecA antibody (bottom panel) revealing an interaction between RecN and RecA. **B)** RecN co-immunoprecipitates with RecA. Immunoprecipitation with an anti-RecA antibody was performed on the WT MG1655 strain and the strain carrying the RecN-flag protein. Samples were blotted with an anti-flag antibody (top panel) or an anti-RecA antibody (bottom panel) revealing an interaction between RecN and RecA. The percentage of IP signal compared to Input signal is indicated

**Supplementary Table 1**

Statistical significance (t-test) of the difference between inter-focal distances measured in the WT and *recN* mutant in Fig 4E and 4F.

| | WT | WT + 15 min MMC | WT + 45 min MMC | *recN* | *recN* + 15 min MMC | *recN* + 45 min MMC |
|---|---|---|---|---|---|---|
| WT | | 0.204 | $4.10^{-37}$ | 0.219 | 0.008 | $3.10^{-44}$ |
| WT + 15 min MMC | | | $9.10^{-25}$ | 0.019 | 0.23 | $2.\ 10^{-30}$ |
| WT + 45 min MMC | | | | $5.\ 10^{-42}$ | $6.10^{-19}$ | 0.12 |
| *recN* | | | | | $2.10^{-4}$ | $5.10^{-48}$ |
| *recN* + 15 min MMC | | | | | | $3.10^{-23}$ |
| *recN* + 45 min MMC | | | | | | |

**Supplementary Methods**

**Construction of *recA-mcherry* fusion.**

The strain JC13509 [1] carries a recA-GFP fusion inserted at the recA chromosomal locus. In addition to the C-terminal fusion with GFP, this recA-GFP allele carries two mutations, a recAo1403 operator mutation (T to A) that increases two-fold the basal level of recA transcription and a recA4155 mutation (R28A) that prevents the formation of DNA-independent polar RecA-GFP foci [1]. In a first step, the ssb gene from the plasmid pKD3-ssb-mcherry was replaced by the recAo1403 4155 gene. For this purpose, the recAo1403 4155 was amplified from JC13509 using the following oligonucleotides: 5' AAC CCA CTC GTG CAC GGC GGG AAT GCT TCA GCG GCG A 3' and 5' TTC ATG TTC GAA TGA TGC TCC CAA AAT CTT CGT TAG TTT CTG C 3' and the PCR products were cleaved with ApaLI and BstBI. The restriction product was cloned into pKD3-ssb-mcherry restricted with ClaI and ApaLI, producing the plasmid pKD3-recA-mcherry. In a second step, the recA-mcherry CmR region of pKD3-recA-mcherry was amplified using the following oligonucleotides, which contain homology regions with the chromosomal lacI and lacZ sequences: 3'GCA GCT GGC ACG ACA GGT TTC CCG ACT GGA AAG CGG GCA GTG AGG CGG GAA TGC TTC AGC GGC GA 5' and 3'TCA TCA TAT TTA ATC AGC GAC TGA TCC ACC CAG TCC CAG ACG AAG ATG AAT ATC CTC CTT AGT TCC TA 5'. The PCR product was used to electroporate MG1655 [pKD46] as described [2] creating a *recA-mcherry* fusion gene inserted into the lac operon under the control of its own promoter recAo1403 (strain JJC5789). The presence of the correct insertion was verified by PCR and by sequencing. The presence of polar foci was unexpected since the recAmcherry allele carries the R28A mutation that prevents recA-GFP polar foci formation [1]. We demonstrated that increased number of polar foci is the consequence of co-expression of recA-mCherry and wild type RecA.

**Ori/ter quantification by qPCR assay on *dnaAts* strains**

An overnight culture was diluted 1:200 in minimum medium A supplemented with 0.2% casamino acids and 0.25% glucose. Cells were grown at 30°C until $OD_{600}$~0.2 and immediately placed at 42°C for inactivation of DnaA. At the same time, 10µg/ml of MMC was added or not. 1,5ml of cells was flash frozen at each time point and genomic DNA was extracted as described above. qPCR was performed on a MyiQ Biorad lightcycler qPCR machine. Results are expressed as the difference between ori Ct and ter Ct.

**Co-immunoprecipitation experiment**

An overnight culture of MG1655 wild type strain and a strain carrying the RecN protein tagged with a flag peptide were diluted 1:200 in Minimum Media A supplemented with 0.2% casamino acids and 0.25% glucose. The cells were grown to an $OD_{600nm}$ of approximately 0.2. Cells were then treated with 10 µg/ml MMC for 30min and pelleted at 4°C for 10min. Cells were washed 3 times with ice cold 1X PBS. Pellets were resuspended in 300µl of lysis buffer (10 mM Tris·HCl (pH 8.0), 150 mM NaCl, 10mM EDTA, 2% (vol/vol) Triton X, 0.2% SDS and Complete protease inhibitor mixture EDTA-free (Roche)) and sonicated 5 times for 30" on and 30" off using a bioruptor sonicator from Diagenode. Cells were pelleted and supernatant was incubated ON at 4°C with an anti-flag antibody (Sigma F3165) (1:300) or anti-RecA antibody (abcam 63797) (1:300). The next day, 100 µl of magnetic anti-proteinA beads were placed on the dynamag and beads were separated from the buffer. The antibody-lysate mix was added to the beads and incubated for 2h at RT. Lysate was then placed on the dynamag and washed three times with 1X PBS. Elution was performed with an SDS buffer (2% SDS, 100 mM Tris-HCl, 10% glycerol and 0.5mM EDTA).

Western blot was done on RecNflag and MG1655 samples using an anti-flag antibody 1:500 (sigma) for the RecN-flag and RecA IP.

Western blot was done on RecNflag and MG1655 samples using an anti-RecA antibody 1:1000 (abcam) for the RecA and RecNflag IP.

**Construction of LoxP strains**

The wild type strain was E. coli K12 MG1655 (ΔlacMluI). The LacloxP cassette was constructed by the integration of a double stranded oligonucleotide 50-

CGTAATAACTTCGTATAATGTATGCTATACGAAGTTATGGATCCCCGGGTACCGAGCTCATAACTTCGTATAATGTATGCTATACGAAGTTATCCTA-30 into the ClaI restriction site of the lacZ gene. The LacloxP cassette was integrated in the chromosome, using a vector derived from pKD4 called pGBKD3-Laclox [3]. These vectors contained the LacloxP cassettes adjacent to the chloramphenicol resistance gene. Laclox::Cm was inserted into the intergenic regions of non-essential genes using the standard 'lambda red' technique [2]**.** Expression of the Cre recombinase was driven by a pSC101 carrying the arabinose-inducible Cre gene derived from pFX465, kindly given by FX Barre. The lacloxP::Cm cassettes inserted at the different chromosome sites were then moved into different strain backgrounds by using P1 transductions.

**Construction of microscopy strains**

To integrate the *parS* sequence in the chromosome of the MG1655 strain, we used a vector derived from pKD4 [2] called pGBKD3-parS pMT1 [4]. pGBKD3- parS pMT1 contains the *parS* sequence from the pMT1 plasmid [5] adjacent to the chloramphenicol resistance gene. All the tags were inserted in intergenic regions of non-essential genes [6]. The expression of the ParB–YGFP fusion protein is driven by the pFH2973 plasmid [5] (Supplementary Table 2).

Supplementary references


1. Renzette, N. *et al.* Localization of RecA in Escherichia coli K-12 using RecA-GFP. *Mol. Microbiol.* **57,** 1074–1085 (2005).
2. Datsenko, K. A. & Wanner, B. L. One-step inactivation of chromosomal genes in Escherichia coli K-12 using PCR products. *Proc. Natl. Acad. Sci. U. S. A.* **97,** 6640–6645 (2000).
3. Lesterlin, C., Gigant, E., Boccard, F. & Espéli, O. Sister chromatid interactions in bacteria revealed by a site-specific recombination assay. *EMBO J.* **31,** 3468–3479 (2012).
4. Espéli, O. *et al.* A MatP-divisome interaction coordinates chromosome segregation with cell division in E. coli. *EMBO J.* **31,** 3198–3211 (2012).

5. Nielsen, H. J., Ottesen, J. R., Youngren, B., Austin, S. J. & Hansen, F. G. The Escherichia coli chromosome is organized with the left and right chromosome arms in separate cell halves. *Mol. Microbiol.* **62,** 331–338 (2006).
6. Espeli, O., Mercier, R. & Boccard, F. DNA dynamics vary according to macrodomain topography in the E. coli chromosome. *Mol. Microbiol.* **68,** 1418–1427 (2008).
